# Predictors for Therapy Response to Intrathecal Corticosteroid Therapy in Multiple Sclerosis

**DOI:** 10.3389/fneur.2019.00132

**Published:** 2019-02-22

**Authors:** Katja Vohl, Alexander Duscha, Barbara Gisevius, Johannes Kaisler, Ralf Gold, Aiden Haghikia

**Affiliations:** Department of Neurology, Ruhr-University Bochum, St. Josef-Hospital, Bochum, Germany

**Keywords:** multiple sclerosis, disease progression, intrathecal corticosteroid therapy, clinical predictor, cerebrospinal fluid

## Abstract

**Objective:** The autoimmune disease Multiple Sclerosis (MS) represents a heterogeneous disease pattern with an individual course that may lead to permanent disability. In addition to immuno-modulating therapies patients benefit from symptomatic approaches like intrathecal corticosteroid therapy (ICT), which is frequently applied in a growing number of centers in Germany. ICT reduces spasticity, which elongates patient's walking distance and speed, thus improves quality of life.

**Methods:** In our study we set out to investigate cerebrospinal fluid (CSF) parameters and clinical predictors for response to ICT. Therefore, we analyzed 811 CSF samples collected from 354 patients over a time period of 12 years. Patients who received ICT were divided in two groups (improving or active group) depending on their EDSS-progress. As control groups we analyzed data of ICT naïve patients, who were divided in the two groups as well. Additionally we observed the clinical progress after receiving ICT by comparison of patients in both groups.

**Results:** The results showed clinical data had a significant influence on the probability to benefit from ICT. The probability (shown by Odds Ratio of 1.77–2.43) to belong to the improving group in contrast to the active group is significantly (*p* < 0.0001) higher at later stages of disease with early disease onset (< 35 years, OR = 2.43) and higher EDSS at timepoint of ICT-initiation (EDSS > 6, OR = 2.06). Additionally, we observed lower CSF cell counts (6.68 ± 1.37 μl) and lower total CSF protein (412 ± 18.25 mg/l) of patients who responded to ICT compared to patients who did not (*p* < 0.05). In the control group no significant differences were revealed. Furthermore analyses of our data revealed patients belonging to the improving group reach an EDSS of 6 after ICT-initiation less often than patients of the active group (after 13 years 39.8% in the improving group, 67.8% in the active group).

**Conclusion:** Our study implies two relevant messages: (i) although the study was not designed to prospectively assess clinical data, in this cohort no severe side effects were observed under ICT; (ii) disease onset, EDSS, CSF cell count, and total protein may serve as predictive markers for therapy response.

## Introduction

Multiple Sclerosis (MS) is one of the most common non-traumatic neurological diseases of young adults (1). Besides the immunological component of MS, axonal damage is a pathological hallmark of disease, which causes permanent disability (2). Despite already existing and approved immuno-modulating therapies for MS, chronic disability, and disease progression caused by neurodegeneration still pose a therapeutic challenge. More than 80% of patients afflicted by MS suffer from spasticity during disease, subsequently leading to critical impairment of daily life routine in 30% of these patients (3), i.e., reduced or diminished walking ability. After insufficient response to first-line (oral) antispastic therapies, intrathecal corticosteroid therapy (ICT) is an adjuvant option to reduce permanent disability (3). Several studies have previously shown the efficacy of ICT in various cohorts (3, 4). ICT can reduce the Expanded Disability Status Scale (EDSS), elongates walking distance and increases walking speed (3). Moreover, ICT improves effectively neuropathic pain which is caused by disease activity (5). In summary, ICT is stated as a safe and effective option for the reduction of disability (6). Additionally, ICT has a beneficial impact on bladder function (3) and generally improves quality of life in responsive patients. Thus, ICT is a promising option to slow down disease progression and reduce permanent disability. However, aside from the clinical improvement no stratifying markers for therapy response/non-response are available so far. Hence, the aim of our study was to identify possible CSF markers that may predict the individual response to ICT. CSF, due to its proximity to MS pathomechanism, has been shown to be a suitable biocompartment, e.g., for epigenetic markers (7), that is usually not affected by systemic metabolic processes derived from the peripheral blood (8).

In our department, we have established (4, 5) and performed ICT for decades. Patients usually receive a cumulative dose of 40–200 mg triamcinolone-acetonide (Volon A) via 1–3 injections (every other day) every 3 months on average in an individualized manner (9). With this study we addressed the following questions: (I) Are there any differences in standard CSF parameters to distinguish between response and non-response to ICT?; and (II) which clinical parameters indicate a beneficial response to ICT?

## Methods

Retrospective data from patients assessed during clinical routine at the Department of Neurology of the Ruhr-University Bochum, St. Josef-Hospital since 2005 were considered for analysis. CSF analysis for possible surrogate markers was approved by the ethics committee of the Department of Medicine at the Ruhr-University Bochum (registration number 4493-12). The mean observation period per patient comprised to 2.58 ± 2.51 years. The distribution of the observation intervals is shown in Table 1. We analyzed a total of 811 CSF samples from 354 different patients. All study patients had been diagnosed with MS according to the McDonald criteria (10) including all different disease subtypes, i.e., relapsing remitting (RR), secondary progressive (SP), and primary progressive (PP). Only those patients who had been unstable with other MS medication for at least 6 months and showed insufficient response to oral first line antispastic therapies received ICT. Written and informed consent of all patients was obtained before initiation of ICT. We screened 206 patients (with 508 CSF analyzed samples) who had received ICT at least at one time point, and 148 patients (with 303 CSF analyses) who had been ICT naïve. Patients and CSF values were included in this study fulfilling the mentioned criteria. Patients were followed longitudinally for their EDSS as well as their CSF.

**Table 1:** Observation Interval of ICT patients; number of values and patients of the observation intervals.

**Observation interval**	**Number of patients**	**Number of CSF values**
Less than 1 year	6	26
1–2 years	93	127
2–3 years	50	75
3–4 years	37	50
4–5 years	34	54
5–6 years	31	66
6–7 years	18	51
7–8 years	15	47
8–9 years	22	81
9–10 years	20	72
10–11 years	11	63
11–12 years	10	51
More than 12 years	7	48

CSF assessment and analysis were performed once before the first ICT injection and afterwards as follow up during longitudinal ICT. Lumbar punctures of patients, who did not receive ICT, were part of diagnostic routine, for instance reevaluation of diagnosis or verification of the conversion of oligoclonal bands. Triamcinolon (40–80 mg) was injected directly into the CSF under sterile conditions using an atraumatic needle (4).

Patients were divided into two groups depending on their respective clinical progress mirrored by individual EDSS (11). EDSS was collected routinely in our department, thus it is possible to monitor patient's disability progression retrospectively over a long period of time as it was essential in our study. We defined two groups based on the ratio of first determined EDSS and the mean of all following respective EDSS of the same patient ≥1: improving group—patients, whose EDSS remained stable or decreased over time < 1: active group—patients, whose EDSS increased over time.

Due to the retrospective character of our study, the evaluation of the EDSS in standardized time intervals was not possible. However, the EDSS assessment prior to the first ICT-injection was evaluated in a time range of 1.2 ± 0.24 years, the EDSS post ICT-injection was evaluated in a time range of 1.68 ± 0.26 years. By definition we excluded patients of whom only one EDSS value was available during the observation period, which leads to an impediment for a definitive group assignment. Hence, for final assessment we included 157 patients with 446 CSF samples who received ICT, and additionally 103 patients with 213 CSF analyses who were ICT naïve. Detailed demographic information of patients is shown in Table 2. We compared these two groups separately for patients who received ICT and patients who were ICT naïve, because of the retrospective vs. cross sectional character of data used for the study. Statistical analysis for CSF parameters of these subgroups was performed by Mann-Whitney-*U*-test, after Kolmogorov–Smirnov-Test had ruled out Gaussian distribution. Therefore, we included all baseline data of CSF of all patients included in the improving or active group to represent the long-term changes. Additionally we analyzed the patients mean cell count to show patient's intrapersonal cell count value dependent on individual disease progress and activity. To examine clinical parameters of patients we used fisher's exact test. For the examination of clinical parameters, we defined specific ranges of these parameters: age range at diagnosis and manifestation were set up to 35 years based on epidemiological data suggesting a transition age of RRMS to SPMS at 33 years (12). We stratified EDSS in below and above EDSS 6 (13, 14).

**Table 2:** Demographic parameters of MS patients; demographic data separated for patients with or without ICT and for improving and active group; ^a^in years (mean ± SD); RRMS, relapsing remitting multiple sclerosis; SPMS, secondary progressive multiple sclerosis; PPMS, primary progressive multiple sclerosis; n, number of patients; f, female; m, male.

			***n***	**Sex**	**Current age^**a**^**	**Age at diagnosis^**a**^**	**Disease duration^**a**^**
No intrathecal corticosteroid therapy	Improving group		51	f = 33; m = 18	51.98 ± 11.62	36.18 ± 13.65	15.8 ± 10.48
		RRMS	27	f = 20; m = 7	46.78 ± 11.55	35.7 ± 13.76	11.07 ± 7.67
		SPMS	20	f = 12; m = 8	57.8 ± 8.77	34.65 ± 13.59	23.15 ± 10.69
		PPMS	4	f = 1; m = 3	58 ± 9.42	47 ± 10.89	11 ± 2.16
	Active group		52	f = 30; m = 22	54.13 ± 10.5	37.46 ± 10.16	16.67 ± 8.49
		RRMS	9	f = 3; m = 6	40.11 ± 14.41	29.33 ± 10.56	10.78 ± 6.36
		SPMS	39	f = 26; m = 13	56.85 ± 6.79	38.23 ± 9.19	18.62 ± 8.52
		PPMS	4	f = 1; m = 3	59.25 ± 3.86	48.25 ± 5.19	11 ± 1.41
Intrathecal corticosteroid therapy	Improving group		72	f = 45; m = 27	51.42 ± 10.57	38.42 ± 10.65	13 ± 8.15
		RRMS	35	f = 22: m = 13	46.66 ± 10.19	37.0 ± 11.2	9.66 ± 6.24
		SPMS	31	f = 19; m = 12	57.29 ± 8.2	39.81 ± 10.43	17.48 ± 8.45
		PPMS	6	f = 4; m = 2	48.83 ± 9.83	39.5 ± 8.76	9.33 ± 5.65
	Active group		85	f = 55; m = 30	54.54 ± 10.98	36.52 ± 10.21	18.02 ± 7.86
		RRMS	14	f = 9; m = 5	44.43 ± 12.11	28.14 ± 11.41	16.29 ± 8.79
		SPMS	62	f = 40; m = 22	56.08 ± 8.81	36.85 ± 8.26	19.23 ± 7.13
		PPMS	9	f = 6; m = 3	59.67 ± 14.46	47.22 ± 10.50	12.44 ± 9.15

Kaplan Maier analysis was performed for evaluating risk of disease progression, i.e., disease progression defined as EDSS increase above 6 over time for both ICT receiving groups; statistical analysis was performed by log-rank (Mantel-Cox) test. All statistical data in figures are shown with mean ± SEM, following p were considered as statistically significant: *p* < 0.05, *p* < 0.001, and *p* < 0.0001.

Additionally, we evaluated the effect of patient specific MS medication in combination with or without ICT, i.e., established immuno-modulating therapies used in MS like interferon-beta, glatiramer acetate, fingolimod, dimethylfumarat, azathioprine, mitoxantrone, and natalizumab.

## Results

### CSF Analyses of ICT Patients

**Figure 1:**
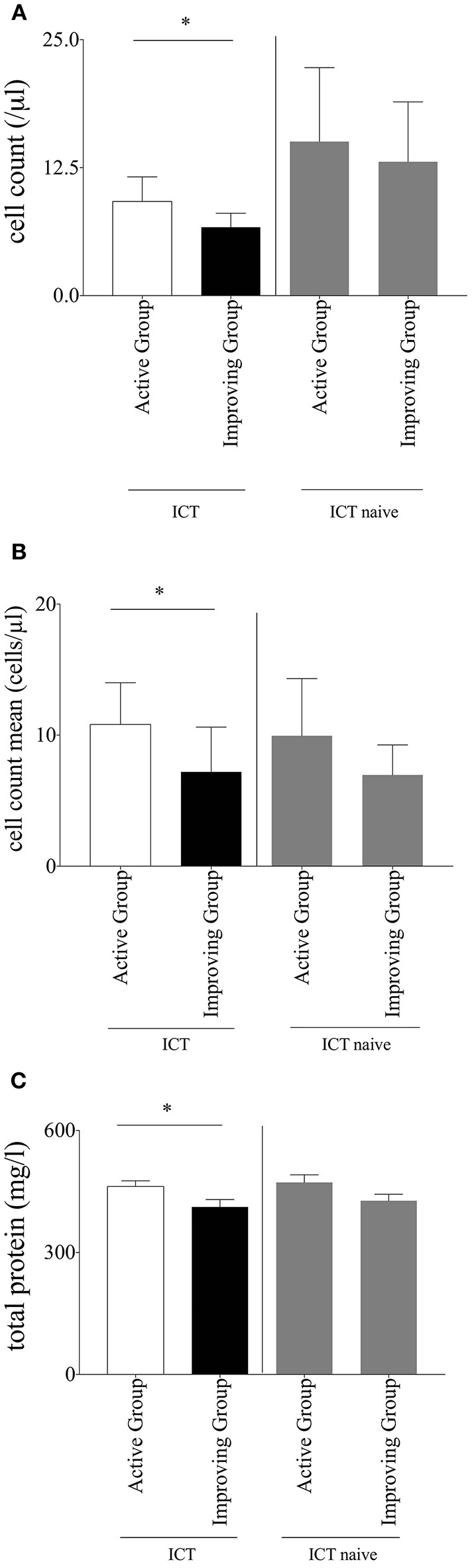
**(A)** Cell count. Baseline cerebrospinal fluid parameter cell count of multiple sclerosis patients within intrathecal corticosteroid therapy (ICT) compared between the subgroups; separate comparison of multiple sclerosis patients without ICT between the two subgroups; Mann–Whitney-*U*-test, mean with SEM, *n* (improving group ICT) = 172, *n* (active group ICT) = 310, *n* (improving group ICT naïve) = 88, *n* (active group ICT naïve) = 137; ^*^p = 0.0442. **(B)** Cell count mean. Mean of cerebrospinal fluid parameter cell count of multiple sclerosis patients within ICT compared between the subgroups; separate comparison of multiple sclerosis patients without ICT between the two subgroups; Mann–Whitney-*U*-test, mean with SEM, *n* (improving group ICT) = 76, *n* (active group ICT) = 59, *n* (improving group ICT naïve) = 46, *n* (active group ICT naïve) = 47; ^*^p = 0.0221. **(C)** Total protein. Baseline cerebrospinal fluid parameter total protein of multiple sclerosis patients within ICT compared between the subgroups; separate comparison of multiple sclerosis patients without ICT between the two subgroups; Mann-Whitney-U-test, mean with SEM; *n* (improving group ICT) = 155, *n* (active group ICT) = 282, *n* (improving group ICT naïve) = 88, *n* (active group ICT naïve) = 137; ^*^p = 0.0014.

Analyzed data of patients who received ICT showed a significant lower absolute cell count in the CSF in the improving group (6.68 ± 1.37 μl) when compared to the active group (9.206 ± 2.39 μl; *p* = 0.04416; Figure 1A). This result was confirmed by observation of the individual patient's mean cell count. Also in this analysis the improving group significantly (*p* = 0.0221) showed a lower cell count mean (7.20 ± 3.41 μl) in comparison to the active group (10.82 ± 3.18 μl; Figure 1B). The amount of total protein in the CSF was significantly lower in the improving group (*p* = 0.0014, improving group 412 ± 18.25 mg/l vs. active group 462.4 ± 14.04 mg/l; Figure 1C). Separately analyzed data of ICT naïve MS-patients did not display significant differences neither in cell count nor in total protein between improving and active group (Figures 1A–C).We detected no significant differences in other standardized CSF parameters including erythrocytes count, glucose, lactate, albumin, and IgG (data not shown).

In addition, we investigated whether patient's individual MS medication had an effect on the specific response to ICT. Therefore, we observed the different immune-modulating therapy options of patients receiving ICT based on their assignment for active and improving group. Since patients only received ICT when they were not stable for at least 6 months with other MS medication, the results confirmed that the effects of ICT were not significantly influenced by the specific MS medication (data not shown).

### Clinical Parameters for a Response to ICT

**Figure 2:**
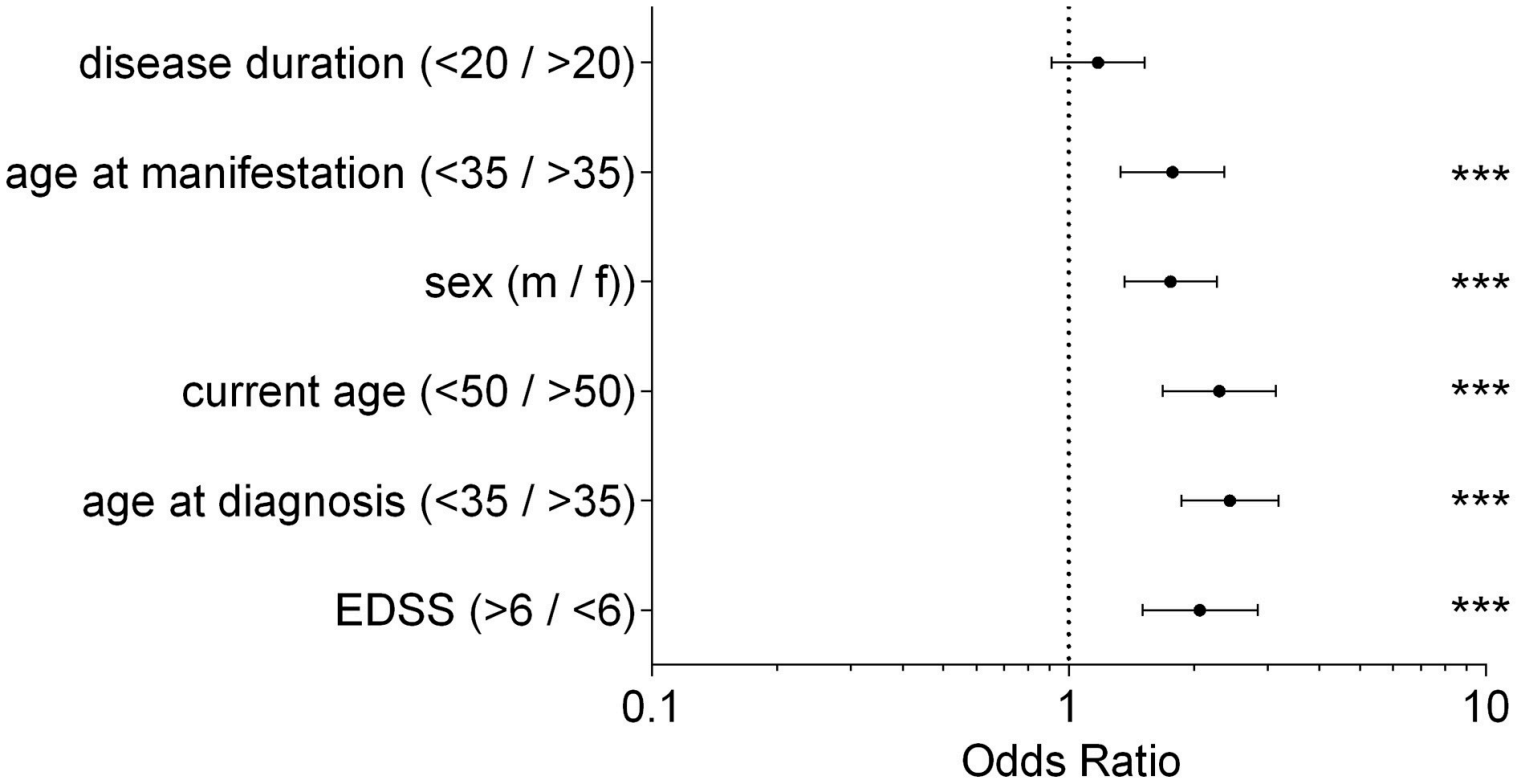
Clinical Parameter; Odds Ratio for each clinical parameter of patients receiving ICT between improving group and active group; fisher's exact test, ^***^p < 0.0001; disease duration (*p* = 0.2383); disease duration/current age/age at manifestation/age at diagnosis in years; EDSS, expanded disability status scale; m, male; f, female.

The analysis of clinical data revealed that an EDSS > 6 at the first injection of ICT correlated with an increased probability to benefit from ICT (OR = 2.06; 95% CI from 1.5 to 1.75; *p* < 0.0001). Additionally, patients below age of 35 years at first diagnosis of MS benefited most from ICT. In contrast to patients older than 35 years, the probability to belong to the improving group was more than twice as high (OR = 2.43, 95% CI from 1.86 to 3.18, *p* < 0.0001). We found similar results for the individual age at first manifestation of the disease (OR = 1.77, 95% CI from 1.33 to 2.36; *p* < 0.0001).The results showed that an age < 50 years currently receiving ICT was accompanied with an increased probability to belong to the improving group (OR = 2.29, 95% CI from 1.68 to 3.14; *p* < 0.0001). Examining the influence of the patients' sex regarding therapy response, male patients responded with a higher probability to ICT than female patients (OR = 1.79, 95% CI from 1.36 to 2.27; *p* < 0.0001). We observed no significant difference between the analyzed groups when assessing disease duration before the start of ICT (OR = 1.18, 95% CI from 0.91 to 1.52; *p* < 0.0001). Results are displayed in Figure 2.

### Time to Reach EDSS 6

**Figure 3:**
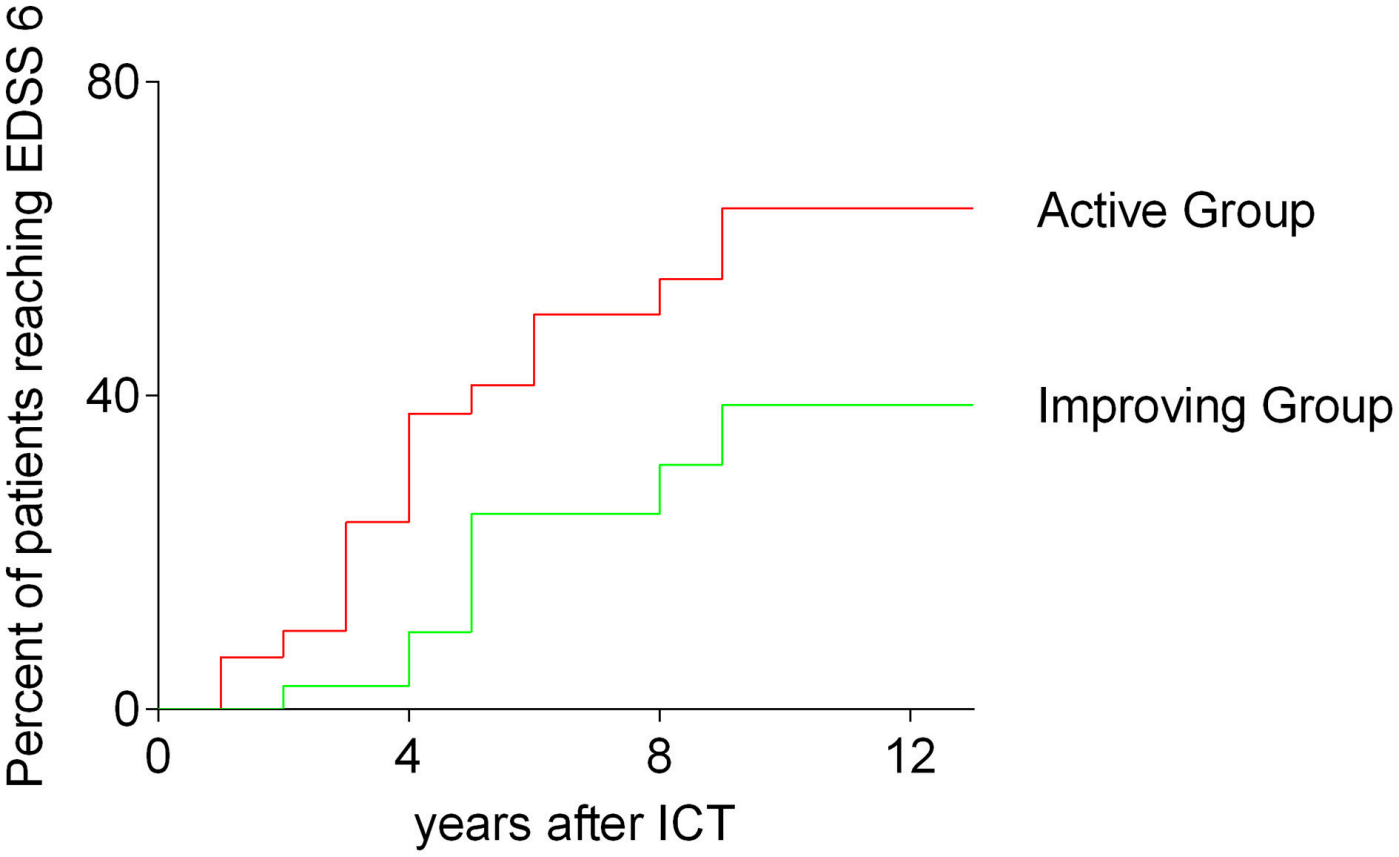
Time to reach EDSS 6; survival curves before reaching an EDSS of 6; all analyzed patients receiving ICT; comparing improving group and active group, Kaplan–Meier survival analysis; log-rank (Mantel-Cox) test, *p* = 0.0357; ICT, intrathecal corticosteroid therapy; EDSS, expanded disability status scale.

We compared both, the improving and the active group of patients receiving ICT in respect to the time of reaching an EDSS of 6. 13 years after first injection of ICT 39.8% of patients in the improving group reached an EDSS of 6, whereas 67.8% of patients belonging to the active group reached an EDSS of 6 [*p* = 0.0357; log rank (Mantel-Cox) test]. The results are shown in Figure 3.

## Discussion

Effective treatment of MS disease progression is one of the major unmet needs in the field of MS therapy. Several studies have demonstrated the safety and efficacy of ICT in MS (3, 4). However, based on its mode of application ICT is not widely used outside German-speaking countries. Also due its invasive nature of application a placebo/sham controlled randomized trial would be un-ethical. The diagnostic value of CSF analysis, however, is being increasingly appreciated even in the age of revised McDonald criteria (10). Our study comprised a cohort of over 200 MS patients with ICT and additionally 148 MS patients without ICT, whose datasets were collected over a time period of 12 years. The weakness of the study is its retrospective nature. However, we could demonstrate a significantly lower level of total protein and cell count in the CSF of patients responding to ICT in a large longitudinally assessed cohort. In accordance with previous studies (3, 15) no severe side effects were observed. Nonetheless, a marker—ideally derived from neurological laboratory routine diagnostic like CSF cell number or total protein—that stratifies responders and non-responders to ICT, may help to assign patients to this therapy. In accordance with previous studies we could stratify clinical data for a positive therapy response to ICT (16).

Our data suggest that patients benefit most from ICT, when (i) diagnosis (and manifestation) was at a younger age (below 35 years of age); (ii) they are younger than 50 years while receiving ICT; (iii) their EDSS is higher than 6 at start of ICT; (iv) when they are male.

CSF analysis is used to exclude differential diagnosis (17) and the standard CSF values are considered as not useful as markers for disease progression or therapy response (8, 18, 19). Among other immunological effects, systemic glucocorticosteroid application has been shown to decrease the number of leucocytes in CSF and to stabilize the blood-brain-barrier (20–22). It is suggested, that low numbers of CSF cells could indicate a beneficial effect on MS progression (20, 23). Furthermore, a dysfunction of the blood-brain-barrier implies a modified expression and secretion of potentially inflammatory mediators in the CSF, e.g., cytokines (24). ICT is an adjuvant option to reduce spasticity and improve patient's motor disabilities, i.e., elongation of walking distance (4). Furthermore, our retrospective data suggest that ICT may have a positive impact on disability progression.

However, the main weakness of this study is its retrospective nature, particularly regarding the clinical data. Hence, the decision whether ICT is used or not is based on an individual risk and benefit assessment. Our data point to a CSF standard assessment, that may serve as a potential tool of predicting therapy response in context of ICT.

## Author Contributions

KV and AD conducted and analyzed the data and wrote the manuscript. BG and JK assessed CSF and edited the manuscript. RG designed the study and edited the manuscript. AH designed and supervised the study, analyzed the data, and edited the manuscript.

### Conflict of Interest Statement

The authors declare that the research was conducted in the absence of any commercial or financial relationships that could be construed as a potential conflict of interest.

## Abbreviations

MS: Multiple Sclerosis
ICT: intrathecal corticosteroid therapy
EDSS: Expanded Disability Status Scale
CSF: cerebrospinal fluid
RRMS: relapsing remitting MS
SPMS: secondary progressive MS
PPMS: primary progressive MS.
